# UBE4B targets phosphorylated p53 at serines 15 and 392 for degradation

**DOI:** 10.18632/oncotarget.6555

**Published:** 2015-12-10

**Authors:** Cheng Du, Hong Wu, Roger P. Leng

**Affiliations:** ^1^370 Heritage Medical Research Center, Department of Laboratory Medicine and Pathology, University of Alberta, Edmonton, Alberta T6G 2S2, Canada

**Keywords:** p53, E3 ligases, ubiquitination, phosphorylation

## Abstract

Phosphorylation of p53 is a key mechanism responsible for the activation of its tumor suppressor functions in response to various stresses. In unstressed cells, p53 is rapidly turned over and is maintained at a low basal level. After DNA damage or other forms of cellular stress, the p53 level increases, and the protein becomes metabolically stable. However, the mechanism of phosphorylated p53 regulation is unclear. In this study, we studied the kinetics of UBE4B, Hdm2, Pirh2, Cop1 and CHIP induction in response to p53 activation. We show that UBE4B coimmunoprecipitates with phosphorylated p53 at serines 15 and 392. Notably, the affinity between UBE4B and Hdm2 is greatly decreased after DNA damage. Furthermore, we observe that UBE4B promotes endogenous phospho-p53(S15) and phospho-p53(S392) degradation in response to IR. We demonstrate that UBE4B and Hdm2 repress p53S15A, p53S392A, and p53-2A(S15A, S392A) functions, including p53-dependent transactivation and growth inhibition. Overall, our results reveal that UBE4B plays an important role in regulating phosphorylated p53 following DNA damage.

## INTRODUCTION

The p53 tumor suppressor is a crucial regulator in response to various stress signals, such as DNA damage, hypoxia and abnormal oncogenic events [1–4]. Having a short half-life, the p53 protein is kept at a low, often undetectable level by rapid degradation in a normal cell mainly through the ubiquitin-proteasome system [5, 6]. Several E3 ubiquitin ligases for p53 have been identified, including Hdm2 (Mdm2 in mouse), Pirh2, Cop1, CHIP and UBE4B [7–11]. Hdm2 is known to be a critical negative regulator of p53 [12–15]. Deletion of Mdm2 in the mouse results in a lethal phenotype in the embryo. However, this early embryonic death of Mdm2-null mice is rescued by further deletion of the *p53* gene, thus indicating the importance of the negative regulatory function of Mdm2 on p53 during development [16, 17]. The human UBE4B is a mammalian homolog of the protein UFD2 found in *S.* cerevisiae [18, 19]. Yeast UFD2 is required for a novel enzymatic activity in ubiquitin chain assembly, and was the first known E4 ubiquitination factor [20]. The deletion of Ube4b in the mouse results in very early embryonic lethality because of marked apoptosis [21]. Polyubiquitination activity for the E4 substrate is greatly reduced in Ube4b^−/−^ mouse embryonic fibroblasts (MEFs) [21]. UBE4B is essential for Hdm2-mediated p53 degradation [11]. UBE4B mediates p53 polyubiquitination and degradation as well as inhibits p53-dependent transactivation and apoptosis [11]. By contrast, Pirh2, Cop1 and CHIP trigger the degradation of p53 independent of Hdm2 [8–10].

p53 is modulated through various post-translational modifications, including phosphorylation, acetylation, ubiquitination, methylation and sumoylation [22]. Post-translational modification is important for regulating the function of p53 [5, 23]. Phosphorylation of p53 at several serine and/or threonine residues has been shown to occur after cells respond to DNA damage. Specifically, serine 15 can be phosphorylated after exposure to gamma irradiation (IR), UV and cadmium [23–27]. Phosphorylation of p53 at serines 20, 37 and 392 could occur after both IR and UV radiation [28–30]. It has been shown that phosphorylation on N-terminal residues, particularly at serines 15 and 37, is believed to induce the disruption of the p53-Hdm2 complex, resulting in the stabilization of p53 [24]. Phosphorylation of p53 at the C-terminal serine 392 (serine 389 in mice) may enhance the specific DNA binding of p53 *in vitro* [31]. Additionally, this phosphorylation event could promote the ability of p53 to suppress cell growth [32–34]. Mice expressing the S389A protein showed bladder tumor development [35].

Here, we report that UBE4B interacts with phosphorylated p53 at serines 15 and 392, and promotes phospho-p53(S15) and phospho-p53(S392) degradation. We observe that the level of UBE4B in the nucleus was significantly decreased in response to ionizing irradiation (IR). In contrast, the level of Hdm2 was increased in the nucleus. Notably, the affinity between UBE4B and Hdm2 is greatly decreased following DNA damage. Our findings shed light on how phosphorylated p53 is regulated in response to DNA damage.

## RESULTS

### p53 phosphorylation and the responses of E3 ligases to DNA damage are different

**Figure 1:**
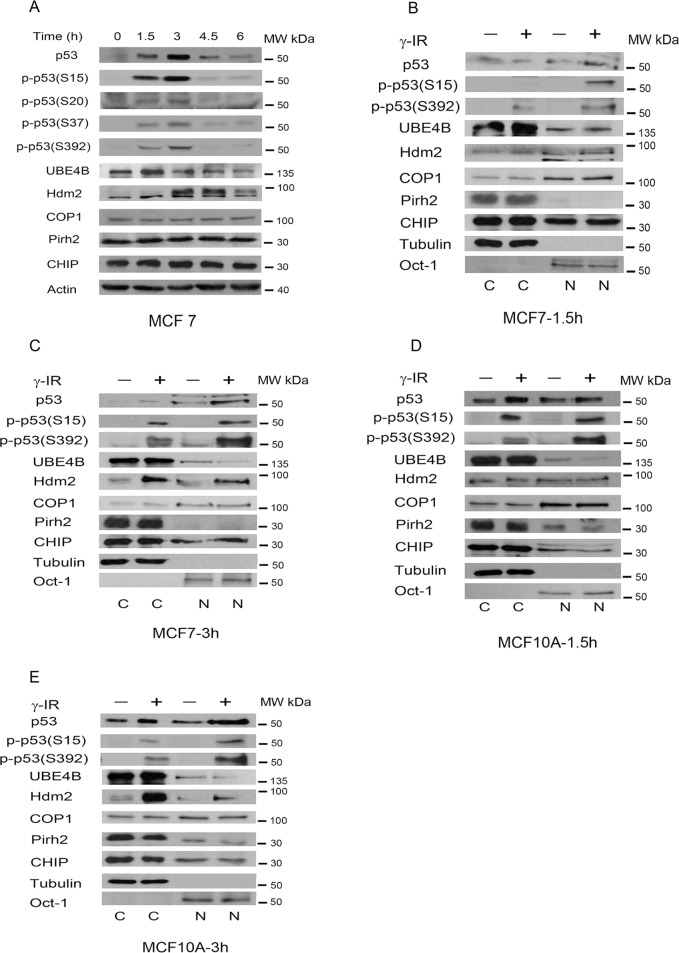
p53 phosphorylation and subcellular localization of various E3 ligases in response to IR **A.** MCF7 cells were treated with 6 Gy of γ-irradiation (IR). Cells were collected at different time points following IR. Phosphorylated-p53 and E3 ubiquitin ligase-specific antibodies were used for western blotting as indicated. **B.** and **C.** MCF7 cells were treated with 6 Gy of IR. Cytoplasmic and nuclear extracts were prepared at 1.5 h (B) and at 3 h (C) after IR (Fermentas, USA). All of the extracts were subjected to western blot analysis using anti-p53 (Pab1801)-, anti-UBE4B (BD)-, anti-Hdm2 (2A10)-, anti-Cop1-, anti-Pirh2-, anti-CHIP (H-231)-specific antibodies. Tubulin was used as the marker for the cytoplasmic (C) fraction, and Oct-1 was used as the marker for the nuclear (N) fraction. **D.** Similar to (B) except that MCF10A cells were used. The cell extracts were prepared at 1.5 hours. **E.** Similar to (C) except that the cell extracts were prepared at 3 hours.

Gamma rays are widely used for cancer treatment. The p53 tumor suppressor protein is activated after exposure to ionizing irradiation (IR) [36]. To study the kinetics of UBE4B, Hdm2, Pirh2, Cop1 and CHIP induction in response to p53 activation, MCF7 cells (a breast cancer cell line) harboring wild-type p53 were treated with IR (6 Gy) for the indicated periods of time. The levels of p53 and UBE4B proteins were increased at 1.5 hours after IR treatment, and the Hdm2 protein level was increased at 3 hours (Figure 1A). Interestingly, total UBE4B levels seem to be decreasing to levels below background at longer times after irradiation (3, 4.5, 6 h). Consistent with the previous report [8], we did not detect any increase in the level of Pirh2 protein in MCF7 cells following DNA damage. No increase in the levels of Cop1 and CHIP was detected in MCF7 cells. Additionally, increased levels of phosphorylated p53 proteins (S15, S20, S37, and S392) were detected at 1.5 hours, reaching a higher peak at 3 hours. We could not detect other signals of phosphorylated p53 (S6, 9, and 46, as well as Thr1; data not shown).

Phosphorylation of p53 is a key mechanism responsible for the activation of its tumor suppressor functions in response to DNA damage or other cellular stresses. One possible explanation is that post-translational modification increases the stability of p53 [5]. To study the subcellular localization of p53, fractionation analysis was employed. MCF7 and MCF10A (a non-tumorigenic breast epithelial cell line) cells were exposed to IR (6 Gy), and nuclear and cytoplasmic extracts were prepared (Fermentas, USA). The nuclear or cytoplasmic fractions were then evaluated using western blot and the nucleus (N)-specific marker Oct-1 antibody or cytoplasm (C)-specific marker tubulin antibody [37]. As shown in Figure 1B, the level of UBE4B was increased only in the cytoplasm but not in the nucleus after 1.5 hours in MCF7 cells. Interestingly, the level of UBE4B in the nucleus was decreased at 3 hours with no change in the cytoplasm (Figure 1C). We observed that the level of UBE4B in the nucleus was significantly decreased at 1.5 hours and 3 hours in MCF10A cells (Figure 1D and 1E). By contrast, the level of Hdm2 in the nucleus was increased at 1.5 hours and 3 hours in MCF7 cells and slightly increased in the nucleus in MCF10A at 3 hours but not at 1.5 hours. Notably, most of the Pirh2 protein was localized in the cytoplasm, and this trend was more obvious in MCF7 cells (Figure 1B and 1C). The proportion of Pirh2 in the nucleus was marginally decreased in MCF-10A cells (Figure 1D and 1E). The levels of Cop1 and CHIP were not changed in the cytoplasm and nucleus (Figure 1B–1E).

Next, immunofluorescence microscopy was employed to illustrate the localization of these E3 ligases in cells. We observed that UBE4B, Cop1, Pirh2 and CHIP mainly localized in the cytoplasm; by contrast, Hdm2 and p53 mainly localized in the nucleus (Supplementary Figure S1). Our data suggest that UBE4B, Cop1, Pirh2 and CHIP could dynamically shuttle between the nucleus and cytoplasm and co-localized with p53 in the nucleus (Supplementary Figure S1).

### UBE4B interacts with phosphorylated p53 at serine 15 and serine 392, and the affinity between UBE4B and Hdm2 was decreased in response to DNA damage

**Figure 2:**
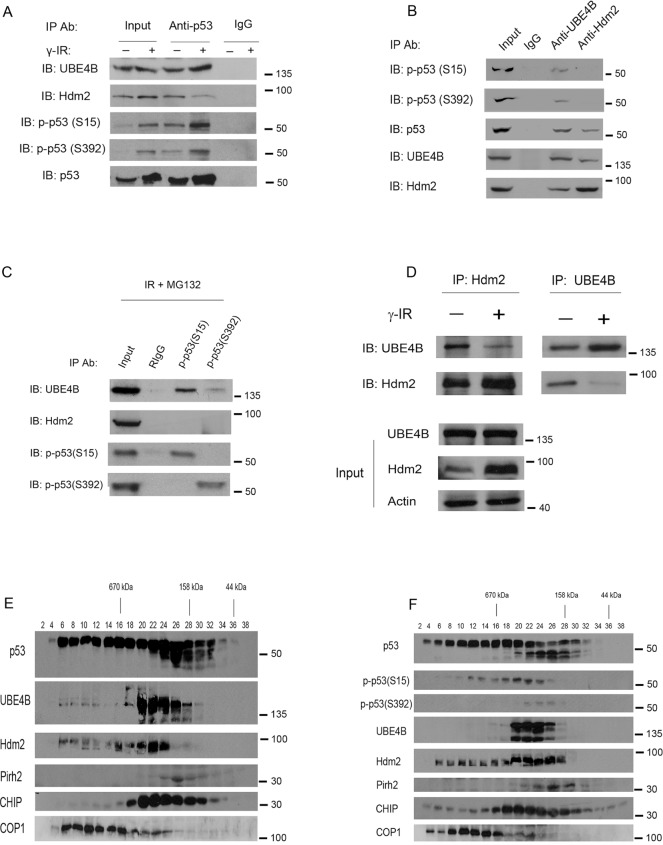
UBE4B interacts with serine 15- and serine 392-phosphorylated forms of p53 **A.** MCF7 cells were treated with or without 6 Gy of IR and harvested at 3 hours after IR. 15% amount of cell lysates were used in the IP as input, whereas 600 μg of total proteins were immunoprecipitated with a p53-specific antibody (DO-1) or mouse IgG and analyzed via western blotting using anti-UBE4B-, anti-Hdm2-, anti-p53S15-, and anti-p53S392-specific antibodies as indicated. **B.** MCF7 cells were treated with 6 Gy of IR and harvested at 3 hours after IR. The cell extracts were immunoprecipitated with anti-UBE4B- and anti-Hdm2-specific antibodies and analyzed via western blotting using anti-p53S15-, p53S392-, anti-p53-, anti-UBE4B-, and anti-Hdm2-specific antibodies as indicated. **C.** A reciprocal IP confirms the interaction between UBE4B and p53(S15) or p53(S392) *in vivo*. MCF7 cells were treated with 6Gy of IR in the presence of MG132 (proteasome inhibitor). Total lysates were immunoprecipitated with p53(S15) or p53(S392) specific antibodies or Rabbit IgG and analyzed by western blot as indicated. **D.** MCF7 cells were treated with or without 6 Gy of IR. The cell extracts were immunoprecipitated with anti-Hdm2 (2A10) or anti-UBE4B as indicated and analyzed via western blotting using anti-UBE4B-, anti-Hdm2-specific antibodies. Direct western blots for UBE4B and Hdm2 are shown in the lower panels. An antibody against β-actin was used as a loading control. **E.** and **F.** Gel filtration of MCF7 lysates. MCF7 cells were left untreated (E) or were treated with 6 Gy of IR (F). The cell extracts were subjected to size-exclusion chromatography on a Superose 6 column. Serial fractions were collected, and proteins were concentrated and analyzed for the presence of p53, Hdm2, UBE4B, Hdm2, Pirh2, CHIP, Cop1, p-p53S15, and p-p53S392 using specific antibodies as indicated.

It was reported that Hdm2 dissociates from p53 after DNA damage because of the phosphorylation of p53 [24]. To determine whether these E3 ligases interact with p53 under stressed or unstressed conditions, coimmunoprecipitation assays (co-IPs) were performed. MCF7 cells were treated with or without IR (6 Gy). The cell extracts were coimmunoprecipitated with a p53 specific-antibody (DO-1) and analyzed using western blotting with antibodies against UBE4B, Hdm2, p-p53 (S15) and p-p53 (S392) as indicated. Consistently, the Hdm2-p53 interaction was slightly reduced; by contrast, the UBE4B-p53 interaction was increased in response to IR (Figure 2A).

Next, we performed co-IP experiments using E3 ubiquitin ligases antibodies to investigate whether they could interact with phosphorylated p53. Phosphorylated p53 at serines 15 and 392 was found to coimmunoprecipitate only with UBE4B; but not with Hdm2 (Figure 2B). In a reciprocal experiment, UBE4B also coimmunoprecipitated with p53(S15) and p53(S392) (Figure 2C). It was reported that UBE4B is essential for Hdm2-mediated p53 polyubiquitination and degradation [11]. To determine the binding ability of UBE4B to Hdm2 in response to IR, MCF7 cells were exposed to IR (6 Gy), and the cells were harvested 3 hours later. The cell extracts were immunoprecipitated with UBE4B-specific and Hdm2-specific antibodies followed by western blot analysis. We found that the interaction between Hdm2 and UBE4B was significantly decreased compared with that of the untreated controls (Figure 2D). These results suggest that DNA damage activates p53 primarily by inhibiting the interaction between Hdm2 and UBE4B.

We then investigated whether these E3 ligases and p53 complex can form in cells. MCF7 cell extracts were fractionated using gel filtration chromatography [11]. As shown in Figure 2E, one major peak was observed at 606–158 kDa (fractions 20–28), although the elution patterns of p53, Hdm2 and Cop1 covered a wider range of fractions than that of UBE4B. These data indicated that these E3 ligases and p53 complex may be formed in the cells. Additionally, our findings revealed that these E3 ligase-phosphorylated p53(S15, S392) complexes can form in the cells (Figure 2F).

### UBE4B promotes phospho-p53(S15) and phospho-p53(S392) degradation

**Figure 3:**
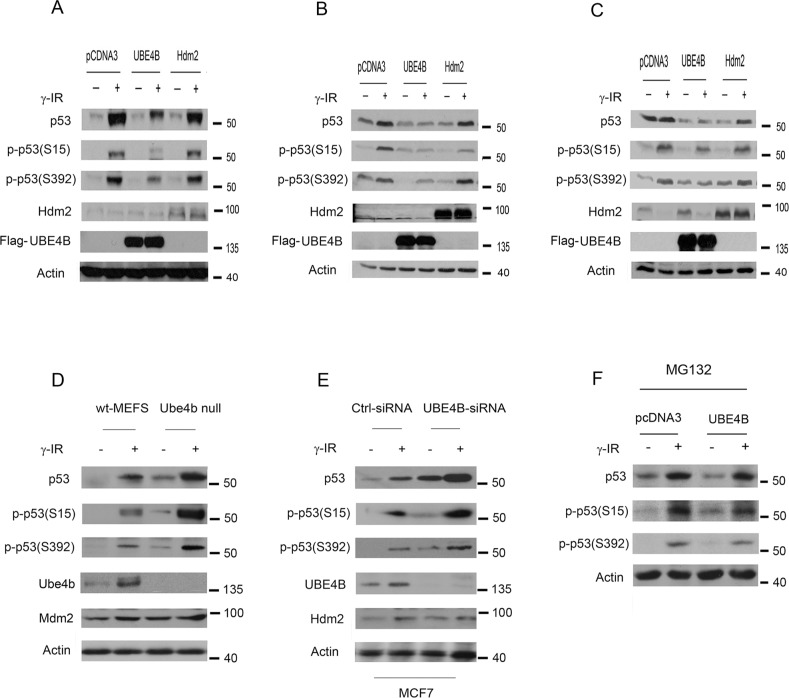
UBE4B targets phosphoserine 15 and 392 p53 for degradation **A.** MCF7 clones stably transfected with plasmids expressing UBE4B, Hdm2, or empty vector (pcDNA3) were treated with or without 6 Gy of IR and subjected to western blotting using anti-p53 (DO-1), anti-p-p53S15, anti-p-p53S392, anti-Hdm2, anti-Flag (M2) for UBE4B. Anti-actin was used as a loading control. **B.** HCT116 cells were transfected with plasmids expressing UBE4B, Hdm2, or empty vector (pcDNA3) and treated with or without 6 Gy of IR. The cell extracts were subjected to western blotting using anti-p-p53S15, anti-p-p53S393, anti-Hdm2, anti-Flag for UBE4B, and anti-actin antibodies. **C.** Similar to (B), except that HEK293 cells were used. **D.** *Ube4b*^−/−^ MEFs or wild-type MEFs were treated with IR (6 Gy) or without treatment. Cells were harvested after three hours treatment and analyzed by western blot with anti-p53 (Pab421), anti-p53S15-, p53S392-, anti-Ube4b-, and anti-Mdm2-specific (MD-129) antibodies. An antibody to β-actin (actin) was used as a loading control. **E.** MCF7 cells were transfected with control-siRNA or UBE4B-siRNA, then these cells were exposed to IR (6 Gy) or without treatment. The amounts of endogenous p53, phospho-p53S15, phospho-p53S392, UBE4B and Hdm2 proteins were determined by western blotting with UBE4B-specific, p53-specific (Pab1801), S15-specific, S392-specific and Hdm2-specific (2A10) antibodies. **F.** MCF7 cells were transfected with plasmids expressing UBE4B or empty vector. Thirty hours later, these cells were treated with IR (6 Gy) or without treatment in the presence of MG132. The cells were harvested after 12 hours and analyzed by western blot using p53-specific (DO-1) or p53S15-specific or p53S392-specific antibodies. An antibody against β-actin was used as a loading control.

Having shown that UBE4B coimmunoprecipitates with phosphorylated p53(S15 and S392), we next asked whether UBE4B could degrade phosphorylated p53. We generated a series of MCF7 clones that stably express UBE4B and Hdm2. These MCF7 stable clones were treated with or without IR (6 Gy). The immunoblot revealed that lower levels of endogenous phosphorylated p53(S15 and S392) in clones expressing UBE4B than clones expressing Hdm2 or the empty vector (Figure 3A). Next, we examined whether transient overexpression of UBE4B reduced the phosphorylated p53 protein levels, as did the MCF7 stable clones expressing UBE4B. HCT116 cells were transiently transfected with plasmids expressing UBE4B, Hdm2, or empty vector. Forty hours later, these cells were treated with or without IR (6 Gy). As shown in Figure 3B, the levels of total p53 were decreased in the presence of UBE4B after IR. The level of phosphorylated p53(S15) was reduced in the presence of UBE4B. Surprisingly, Hdm2 slightly decreased this form of p53 as well. Notably, the level of phosphorylated p53(S392) was significantly decreased in the presence of UBE4B. Similar data were obtained from HEK293 cells transiently overexpressing these E3 ligases (Figure 3C). Moreover, we observed that the levels of total p53 and phospho-p53S15 and S392 in Ube4b null MEFs were greatly increased in response to DNA damage compared with that of wt-MEFs (Figure 3D). Consistently, our findings revealed that the levels of total p53 and phospho-p53S15 and S392 were significantly increased in MCF7 cells depleted UBE4B by siRNA after DNA damage (Figure 3E). To determine whether UBE4B mediates p53S15 or p53S392 degradation via the ubiquitin/proteasome pathway, plasmids expressing UBE4B or empty vector were transfected into MCF7 cells. Thirty hours later, these cells were treated with IR (6 Gy) or without treatment in the presence of a proteasome inhibitor, MG132. The addition of MG132 greatly increased the levels of total p53 or p53S15 or p53S392 in the cells transfected UBE4B compared with the empty plasmid, suggesting that UBE4B promotes p53 or p53S15 or p53S392 degradation via the ubiquitin-proteasome pathway (Figure 3F). Taken together, these data indicated that UBE4B negatively regulates phosphorylated p53 at Ser15 or Ser392 in response to DNA damage.

### Ubiquitination of p53 or p53S15A or p53S392A mediated by UBE4B or Hdm2 after DNA damage is different

**Figure 4:**
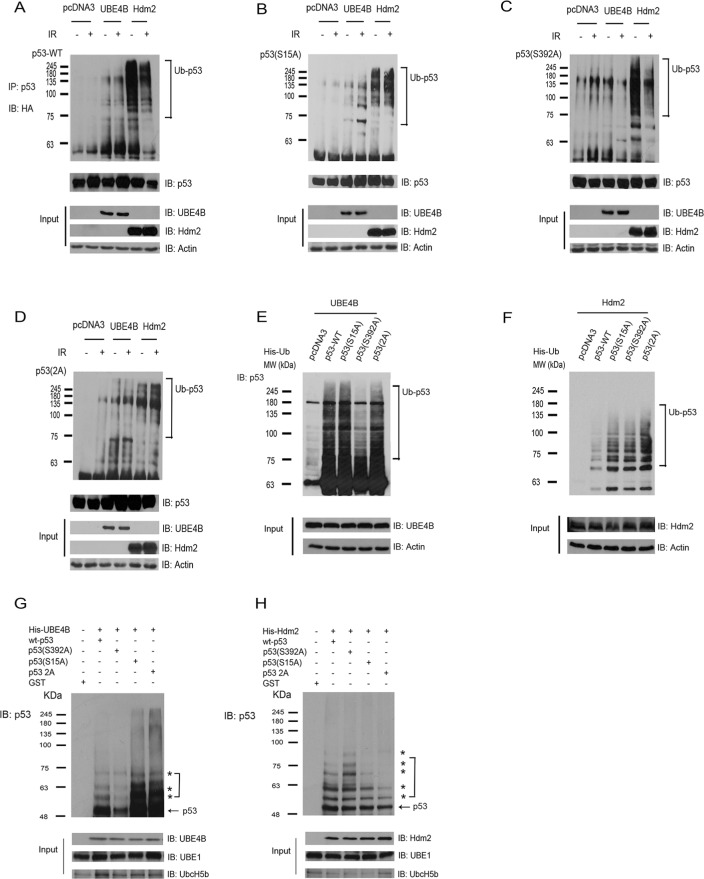
UBE4B and Hdm2 mediate p53 or p53 phosphomutants' ubiquitination *in vivo* and *in vitro* **A.** H1299 cells were co-transfected with HA-tagged ubiquitin and wt-p53 expression plasmids in combination with UBE4B and Hdm2 expression constructs or empty vector (pcDNA3) as indicated. The cells then were treated with or without 6 Gy of IR. Cell lysates were immunoprecipitated with a p53-specific antibody (Pab1801) and analyzed using western blotting with antibodies against HA (top) or p53 (DO-1, bottom). Direct western blots for UBE4B, Hdm2 and actin are shown in the lower panels. **B.** Similar to (A), except that p53(S15A) was used. **C.** Similar to (A), except that p53(S392A) was used. **D.** Similar to (A), except that p53-2A (S15A, S392A) was used. **E.** H1299 cells were cotransfected with plasmids expressing His-ubiquitin, wt-p53 or p53S15A or p53S392A or p53-2A (S15A, S392A) together with UBE4B. His-ubiquitinated proteins were isolated from denatured whole cell extracts and analyzed via western blotting using a p53-specific antibody (DO-1). Direct western blots for UBE4B and actin are shown in the lower panels. **F.** Similar to (E), except that Hdm2 was used. **G.** His-UBE4B or **H.** His-Hdm2 was evaluated for its capacity to ubiquitinate purified GST-wtp53 or GST-p53S15A or GST-p53S392A or GSTp53-2A (S15A, S392A) *in vitro* and was analyzed using immunoblotting with antibodies directed against p53 (DO-1). Direct western blots for UBE4B, or Hdm2, UBE1 and UbcH5b are shown in the lower panel.

To investigate whether these E3 ligases affect the ubiquitination of phospho-p53(S15) and phospho-p53(S392), we examined p53 ubiquitination *in vivo* using two different experiments. Serines 15 and 392 in p53 were substituted by alanine, resulting in the partial disability of phosphorylation. We co-expressed HA-tagged ubiquitin, wt-p53, p53 variants and UBE4B or Hdm2 in H1299 cells. The cells then were treated with IR (6 Gy) or without treatment. The cell extracts were immunoprecipitated with a p53-specific antibody (Pab1801) and immunoblotted with an anti-HA antibody to detect ubiquitinated p53 or with a p53-specific antibody DO-1 to detect total p53 (Figure 4A–4D). We observed that DNA damage inhibits wt-p53 or p53(S392) ubiquitination mediated by Hdm2 (Figure 4A, 4C) compared with that of without IR. The ubiquitination of p53(S15A) mediated by UBE4B after IR was increased (Figure 4B). The ubiquitination of p53(S392) mediated by UBE4B was reduced after IR (Figure 4C). Ubiquitination of p53(S15A) mediated by Hdm2 were slightly increased when treated with IR (Figure 4B). No difference was found in the ubiquitination of p53-2A (S15A, S392A) mediated by UBE4B or Hdm2 treated with or without IR (Figure 4D). To provide direct evidence that the modified p53 species corresponds to ubiquitin conjugation, we coexpressed His-tagged ubiquitin and p53, p53S15A, p53S392A, p53-2A, UBE4B or Hdm2 in H1299 cells and isolated His-ubiquitin conjugated proteins under denaturing conditions. After extensive washing, ubiquitinated proteins were eluted and analyzed via western blotting using a p53-specific antibody (DO-1) (Figure 4E–4F). We observed that wt-p53 and p53(S15A) were heavily ubiquitinated in the presence of UBE4B and to a lesser extent in the presence of Hdm2 (Figure 4E–4F). Interestingly, we observed that the ubiquitination level of p53 was decreased with UBE4B co-expression when serine 392 was substituted with alanine, suggesting that serine 392 is very important for UBE4B-induced p53 ubiquitination (Figure 4E).

The experiments described above were performed in living cells that contained endogenous E1, E2, E3 and E4. To determine whether p53(S15A), p53(S392A) and p53(2A) could serve as a substrate for UBE4B or Hdm2-dependent ubiquitination *in vitro*, affinity-purified His-UBE4B or His-Hdm2 proteins was added to bacterial extracts containing recombinant E1 and E2 (UbcH5b), ubiquitin, and purified GST-p53 and GST-p53 variants. Ubiquitination of the purified GST-p53 and GST-p53 variants was evaluated using western blotting. The p53 immunoblot revealed that (**a**) UBE4B or Hdm2 could function as an E3 ligase to promote the ubiquitination of p53 or p53 variants *in vitro*; (**b**) UBE4B or Hdm2 alone mediated mono- or multiple ubiquitination of wt-p53 and p53 variants *in vitro*; **(c)** consistently, we observed that ubiquitination of p53(S392A) mediated by UBE4B was greatly decreased *in vitro*, confirming that UBE4B plays an important role in regulating ubiquitination of phosphorylated p53 at serine 392 (Figure 4G and 4H).

### UBE4B and Hdm2 inhibit p53- or p53S15A- or p53S392A-mediated transactivation and apoptosis

**Figure 5:**
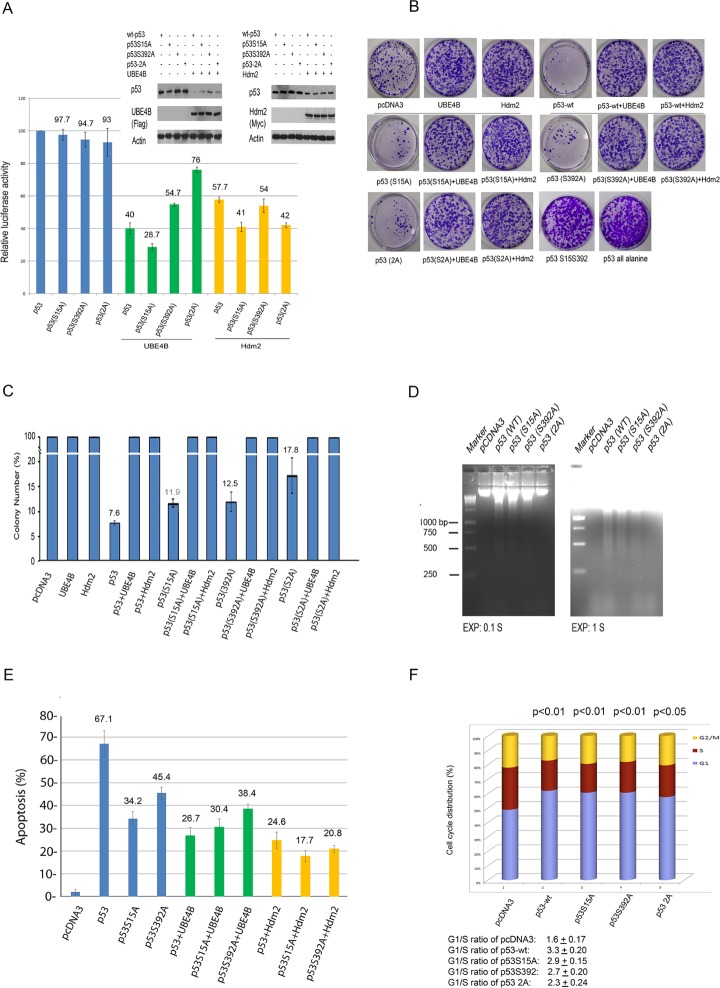
UBE4B and Hdm2 both repress wtp53- and p53 variant-mediated transactivation and growth suppression **A.** H1299 cells were cotransfected with a p21-Luc reporter plasmid and the wt-p53 or p53S15A or p53S392A or p53-2A expression constructs in combination with the Hdm2 or UBE4B expression construct or an empty vector (pcDNA3.1). Luciferase activity was measured. Error bars indicate the S.E.M. (*n* = 3). The relative changes in luciferase activity are shown. Western blot analysis of p53, p53S15A, p53S392A, p53-2A, Hdm2, and UBE4B following transfection was performed using Pab1801 for p53 and p53 variants, an anti-Myc antibody for Hdm2, and M5 for UBE4B. **B.** The effect of UBE4B or Hdm2 on p53 or p53S1A or p53S392A or p53-2A (S15A, S392A) or p53-2S (S15, S392) or p53 all alanine-dependent growth suppression. H1299 cells were stably transfected with pcDNA3-neo-p53 or p53 variants alone, an empty vector, or in combination with UBE4B, or with Hdm2. Cells were plated under G-418 selection 2 days after transfection, and the drug-resistant colonies were stained with crystal violet and enumerated 2 week later. Colony formation in representative dishes of transfected H1299 cells is shown. **C.** Similar to (B), the numbers were the average of three independently transfected plates. Error bars indicate the S.E.M. (*n* = 3). **D.** DNA fragmentation of wt-p53 and p53 variants induced apoptosis in transfected H1299 cells. EXP, exposure time. **E.** H1299 cells were cotransfected with a CD20 expression construct and with pcDNA3-p53 (3 μg) or pcDNA3-p53S15A or pcDNA3-p53S392A and pcDNA3-UBE4B (15 μg), or pcDNA3-Hdm2 (15 μg). The inhibitory effect of UBE4B on p53-dependent apoptosis was determined using annexin V staining of CD20-positive cells and flow cytometry. Error bars indicate the S.E.M. (*n* = 3). **F.** H1299 cells were transfected with empty vector (pcDNA3), wt-p53, p53S15A, p53S392A or p53-2A. The cell profile was analyzed by propidium iodide staining and flow cytometry 40 hours after transfection. G1/S ratio is the ratio of subpopulations and G1-S phase fractions which illustrates the degree of G1 arrest. Each G1/S ratio is presented as indicated. P< 0.01 (2-tailed Student *t* test).

To determine the effect of UBE4B or Hdm2 expression on p53- or p53 variant-mediated transcriptional activation, UBE4B, Hdm2 or empty vector was cotransfected into H1299 cells with wt-p53 or p53S15A or p53S392A or p53-2A (S15A, S392A), together with a luciferase reporter construct (p21-luc) containing the p53 binding site of p21 promoter [8,11]. As shown in Figure 5A, UBE4B and Hdm2 both repressed wt-p53- or p53S15A- or p53S392A-mediated transactivation. Notably, UBE4B strongly inhibited p53S15A-mediated transcriptional activation (Figure 5A). Moreover, we observed that UBE4B showed lesser inhibition of p53-2A mediated transactivation, indicating that p53 at serine 392 is important for the function of UBE4B (Figure 5A). It has been reported serine 15 phosphorylation is important in the regulation of p53 function [27, 38]. We investigated whether UBE4B can interact with p53S15A or p53S392A *in vivo*. p53-null H1299 cells were transfected with plasmids expressing p53S15A and p53S392A, or in combination with Flag-UBE4B, immunoprecipitated with the indicated antibodies, and analyzed by western blots. The data showed that UBE4B coimmunoprecipitated with p53S15A and p53S392A *in vivo* (Supplementary Figure S2A and S2B).

To investigate the biological role of the interaction between UBE4B-p53S15A or UBE4B-p53S392A, we used a long-term colony forming assay to examine cell survival. H1299 cells were transfected with plasmids expressing wt-p53, p53S15A, p53S392A, or an empty vector alone or in combination with UBE4B or Hdm2. Cells were replated at equal numbers and placed under G-418 selection 2 days after transfection, and the drug-resistant colonies were enumerated 2 weeks later. As shown in Figure 5B, wt-p53 dramatically suppressed colony formation compared with the control. As expected, S15A, S392A, and 2A (S15A, S392A) mutants showed slightly reduced activity compared with the wild-type p53 (Figure 5B). We observed that both UBE4B and Hdm2 could inhibit wt-p53- or p53S15A- or p53S392A-mediated cell death (Figure 5C). Apoptosis was examined by the characteristic fragmentation of DNA. The DNA fragmentation assay illustrated that the DNA ladder of the double-mutant p53-2A (S15A, S392A) nearly disappeared, suggesting that the phosphorylation of serine 15 and 392 may be essential for the induction of apoptosis (Figure 5D). Furthermore, we examined whether UBE4B inhibits p53S15A- or p53S392A-dependent apoptosis (wild-type p53 as a control). Transient expression experiments were performed by FACS analysis (using annexin V staining) to determine whether UBE4B expression can rescue cells from p53- or p53S15A- or p53(S392A)-dependent cell death. Consistently, our findings revealed that p53S15A or p53S392A greatly decreased the apoptotic activity of p53, indicating that Ser15 or Ser392 plays an important role in mediating the apoptotic activity of wild-type p53 (Figure 5E). The increase in apoptosis by wild-type p53 could be largely prevented by the co-expression of UBE4B or Hdm2 (Figure 5E). However, UBE4B had little effect on the apoptotic activity of p53S15A or p53S392A. Notably, we observed that Hdm2 further reduced the apoptotic activity of p53S15A or p53S392A (Figure 5E).

To determine the physiological role of p53 at serine 15 or serine 392, we generated p53S15, p53S392 and p53-2S(S15, S392) mutants containing one or two phosphorylatable serine residues, with the remaining serine residues mutated to alanine [S15, or S392, or 2S (S15, S392)], and p53 all alanine (all serine residues have been mutated to alanine) using gene synthesis services (Life Technologies, USA). H1299 cells were transfected with plasmids encoding p53S15, p53S392, or p53-2S (S15, S392). Cells were placed under G418 selection, and drug-resistant colonies were selected two weeks later. As shown in Figure 5B, like p53 all alanine, p53-2S (S15, S392) could not induce cell death. Similar data were obtained for p53S15 and p53S392 (data not shown). It is possible that one or two wild-type p53 phosphorylation sites may not have a detectable effect on p53 activity. We cannot, however, rule out the possibility that other phosphorylation sites of p53 are important for p53-mediated growth suppression.

**Figure 6:**
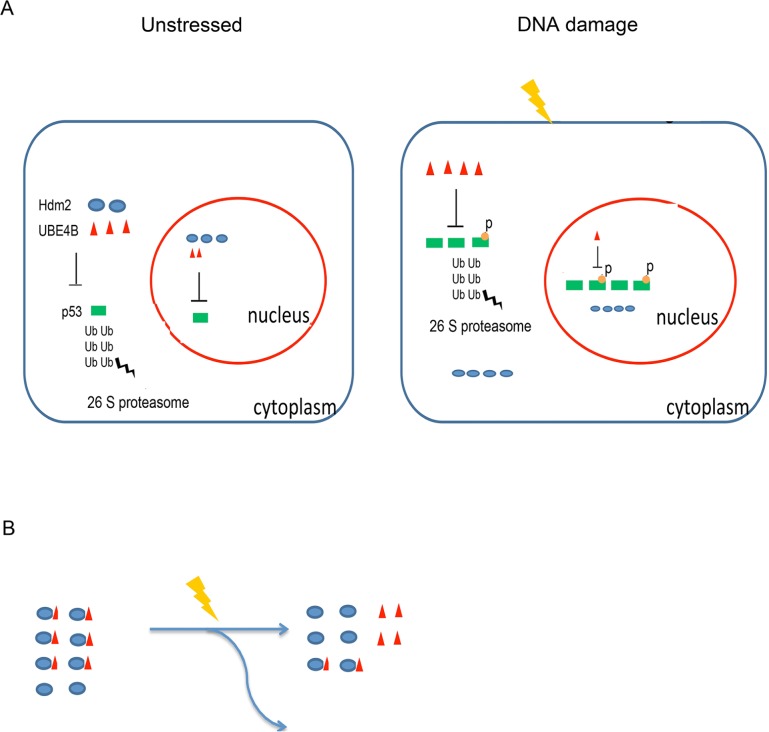
A Model for p53 regulation via UBE4B or Hdm2 under normal or stressed conditions **A.** p53 is kept at a low level in the cytoplasm and nucleus under normal conditions. Following DNA damage, p53 is activated, and some of the protein is phosphorylated and stabilized in the nucleus. The expression level of Hdm2 is also increased, but it cannot degrade post-translationally modified p53. Although UBE4B degrades phosphorylated p53, UBE4B was decreased in the nucleus. **B.** UBE4B dissociates from Hdm2, resulting in the alleviation of p53 degradation. Next, p53 levels started to decrease because of the remaining UBE4B and other E3 ubiquitin ligases in the nucleus.

To investigate whether there is a functional consequence of decreased p21 induction, flow cytometry was performed to analyze the cell cycle arrest function of wild-type p53 and various p53 mutants, or in combination with Hdm2 or UBE4B or Pirh2 or Cop1 or CHIP. As shown in Figure 5F, G1-arrest cells were partly decreased in mutant p53 transfected cells when compared to wild type p53. Surprisingly, we did not detect a significant decrease in cell cycle arrest when cells overexpressed Hdm2, UBE4B, Pirh2, Cop1 or CHIP, suggesting that ectopic expression of these E3 ligases do not appear to have an effect on cell cycle progression (data not shown). Previous studies have demonstrated that siRNA-mediated inhibition of endogenous Hdm2, UBE4B, Pirh2, or Cop1 increases the level of endogenous p53 protein and is accompanied by cell cycle arrest in G1 [39, 11, 8, 9]. The latter finding suggests that these E3 ligases exert a negative control at the level of both protein stability and protein activity. However, we could not obtain opposing effects with overexpression of these E3 ligases. It was reported that Hdm2 accelerated cell cycle progression in human RPMI2650 cells; but not in Saos-2, H1299 and U2OS cells [40]. Therefore, it is possible that the opposing effects with overexpression of these E3 ligases appear to be dependent on cell types. Further experiments are required to address the mechanism. Taken together, our findings led to a model whereby UBE4B and Hdm2 regulate p53 under normal conditions (non-stressed conditions) or in response to DNA damage (Figure 6).

## DISCUSSION

The p53 tumor suppressor plays a critical role in response to various cellular stresses, including DNA damage. Activation and stabilization of p53 lead to growth arrest or apoptosis. Posttranslational modifications involving phosphorylation and acetylation are believed to accumulate, stabilize and activate p53 in the nucleus [5]. A number of studies have proposed explanations for the stability of p53 in response to DNA damage such as (**a**) Hdm2 dissociates from p53 after DNA damage [24]. (**b**) phosphorylation of Hdm2 at serine 395 after gamma irradiation, which alters Hdm2 ligase activity, prevents p53 degradation and export from the nucleus [41]. (**c**) Mdm2 is rapidly degraded by SCF^β-TRCR^ ubiquitin ligase after DNA damage [42]. (**d**) ATM activity switched Hdm2 from a negative to a positive regulator of p53 via the p53 mRNA-Hdm2 interaction [43]. (**e**) Mdm2 Ser394 phosphorylation is required for DNA damage-induced p53 activation [44]. However, the mechanism of phosphorylated p53 regulation is not fully understood. Hdm2 is a critical negative regulator of p53 [7–11]. Surprisingly, Hdm2 mediates mono- or multiple-monoubiquitination of p53 [45–48], but only polyubiquitin chains are efficiently recognized by the proteasome [45–47]. Hdm2 does not polyubiquitinate p53, which suggests that the activity of other ubiquitin ligases is required for Hdm2-mediated p53 degradation [49–52]. In our previous studies [11], we showed that UBE4B is essential for Hdm2-mediated p53 ubiquitination and degradation. In this study, we demonstrated that UBE4B targets p53 phosphorylated at serines 15 and 392 for degradation in response to DNA damage.

We found that the level of UBE4B in the nucleus was decreased significantly in response to IR; by contrast, the level of Hdm2 in the nucleus was increased. We observed that the binding ability of Hdm2 to p53 was decreased in response to gamma irradiation, although the expression level of Hdm2 increased along with p53. Coimmunoprecipitation and gel filtration analysis confirmed that UBE4B interacts with serine 15 and 392 phosphorylated p53 *in vivo*. We showed that UBE4B dissociated from Hdm2 following DNA damage, a finding that may also affect the degradation of p53. Moreover, we observed that UBE4B degrades phosphorylated p53 at Ser15 and Ser392 in different cell lines after IR. Consistently, the levels of total p53 and phospho-p53S15 and S392 after DNA damage were greatly increased when the cells depleted UBE4B by siRNA or/and in Ube4b null MEFs. Notably, we observed that overexpression of Hdm2 decreased the level of p53S15 following IR in HCT116 and HEK293 cells although we were unable to detect the interaction between Hdm2 and p53S15. Future experiments will further investigate the mechanism. In addition, ubiquitination of p53 mediated by UBE4B was substantially decreased when serine 392 was substituted by alanine, whereas the ubiquitination level of phosphorylated wild-type p53 by Hdm2 was alleviated compared with that of the control following DNA damage. We further observed that UBE4B and Hdm2 both repress wild-type p53- or p53S15A- or p53S392A-mediated transcriptional activity and growth suppression.

In our previous studies [11], we demonstrated that **(a)** the basal level of endogenous p53 protein is much higher in mouse Ube4b null MEFs than in parental wild-type MEFs; indicating Ube4b plays a critical role in regulating basal level of p53 in unstressed conditions; **(b)** overexpression of mouse Ube4b decreased the level of p53 in Mdm2 null MEFs, suggesting that the Ube4b-dependent Mdm2-mediated p53 degradation is not absolute. Currently, we showed that UBE4B negatively regulates phospho-p53 (S15 and S392) in response to IR; indicating UBE4B plays an important role in regulating p53 stability following DNA damage. It was reported that Hdm2 dissociated with p53 in response to DNA damage, leading to p53 stabilization. Our findings revealed that UBE4B targets phosphorylated p53 for degradation independently of Hdm2. Recently, the UBE4B-induced degradation of p53 has been confirmed [53, 54]. It was reported that UBE4B is overexpressed in breast cancer [55] and hepatocellular carcinoma [56] and there is an inverse correlation between the overexpression of UBE4B and a decreased p53 level in these tumors [55, 56]. Yang et al reported that CARP1 and CARP2 (caspase 8/10 associated RING proteins) target phosphorylated p53 at serine 20 for ubiquitination and degradation, independent of Hdm2 [57]. They proposed that CARPs may also target phospho-p53 at serine 15 for degradation, however; it's not yet been confirmed [57].

Changing serine 15 to alanine partially reduced the ability of p53 to undergo growth arrest [52, 38]. Additionally, serine 392 may compensate for the functional loss of serine 15 in mutant p53 proteins from human tumors [58]. Here, we show that UBE4B targets phospho-p53 at serine 15 and serine 392 for ubiquitination and degradation. Therefore, our results provide a new concept in which UBE4B plays a significant role in regulating phosphorylated p53 in response to DNA damage.

## MATERIALS AND METHODS

### Cell culture and DNA transfection

Human H1299, HCT116, MCF7 and MCF10A cells were maintained in α-minimal essential medium supplemented with 10% fetal bovine serum. For transient or stable assays, plasmids were transfected using the calcium phosphate method as described earlier [11, 59].

### Plasmids, reagents and antibodies

The p53 mutants S15A, S392A, 2A were generated using the QuickChange site-directed mutagenesis kit (Stratagene, USA). All of the plasmids were verified by sequencing. Antibodies were purchased from the indicated vendors: human p53 (DO-1 or Pab1801 or FL-393; Santa Cruz Biotechnology), Hdm2 (2A10; EMD Biosciences), UBE4B (BD Biosciences or RQ-5 from Santa Cruz Biotechnology), CHIP (H-231; Santa Cruz Biotechnology), Cop1 and Pirh2 (Bethyl Laboratories), phospho-p53 antibodies (#9919; Cell Signaling), Oct-1 (12F11; Santa Cruz Biotechnology), tubulin and actin (Sigma), Flag (M2 monoclonal antibody, Sigma), Myc-specific antibody (9E10; Roche), and HA (12CA5; Roche).

### Gel filtration

Gel filtration was performed as described previously [11] with a few modifications. Cell extracts were prepared in lysis buffer (50 mM Tris-HCl (pH 7.5), 150 mM NaCl, 1% NP40). The same Tris buffer (50 mM Tris-HCl (pH 7.5), 150 mM NaCl) was used for Superose 6 column (GE Healthcare) equilibration and protein elution.

### Coimmunoprecipitation

Cell extracts were prepared in lysis buffer (50 mM Tris-HCl (pH 7.4), 1 mM EDTA, 150 mM NaCl, 0.5% NP40, 1× protease inhibitor (Roche)). The protein concentration was balanced using the Bio-Rad Protein Assay. Approximately 500 μg to 1 mg of the lysates were immunoprecipitated with the indicated antibodies for 1 hour, incubated with protein A/G Plus-agarose (Santa Cruz Biotechnology) beads for 2 hours at 4°C, and then washed three times with washing buffer (50 mM Tris-HCl (pH 7.4), 1 mM EDTA, 150 mM NaCl, 0.2% NP40). The immunoprecipitates were analyzed using western blotting.

### *In vivo* ubiquitination assay

Cells were cotransfected with expression plasmids encoding p53 or p53 variants, HA-tagged ubiquitin, and various E3 ligases. After 40 hours, cells were treated with or without 6 Gy of IR and were immunoprecipitated with the indicated antibodies. Immune complexes recovered with protein A-Sepharose were washed 4 times with RIPA buffer, separated using 10% SDS-PAGE, and analyzed by immunoblotting as described previously [11, 59].

### His-ubiquitin pull-down assay

Cells were transfected with His-tagged ubiquitin and the indicated expression plasmids. Thirty hours after transfection, the cells were resuspended in Buffer A (6 M guanidine-HCl, 0.1 M Na_2_HPO_4_/NaH_2_PO_4_, 10 mM Tris-HCl (pH 8.0); 10 mM imidazole at pH 8.0) and sonicated. Approximately 500 μg of cell lysates were added to 50 μl of equilibrated Ni-NTA agarose and were allowed to incubate for 3 h at room temperature. Beads were then washed once with Buffer A, followed by two washes with **Buffer A/TI** (1 vol of Buffer A, 3 vol of **Buffer TI** [25 mM Tris-Cl, 20 mM imidazole at pH 6.8]), and one wash with Buffer TI; all of washes used 1 ml of the buffer. After extensive washing, the precipitates were boiled with SDS loading buffer and then subjected to SDS-PAGE followed by immunoblotting analysis.

### *In vitro* ubiquitination assay

The *in vitro* ubiquitination assay was performed as previously described [11, 59] with some modifications. For UBE4B- or Hdm2-mediated ubiquitination, rabbit E1 (20–40 ng; Calbiochem), UbcH5b (100 ng; Calbiochem), ubiquitin or His-ubiquitin (5 mg; Sigma), His-UBE4B or His-Hdm2, and GST-p53 or GST-p53S15A or GST-p53S392A or GST-p53 2A were added to the ubiquitination buffer (50 mM Tris-HCl (pH 7.4), 2 mM ATP, 5 mM MgCl_2_, and 2 mM DTT) to obtain a final volume of 30 ml. The reactions were incubated at 30°C for 1.0–1.5 hours. The reactions were stopped with 2× SDS loading buffer, resolved on SDS-PAGE gels, and analyzed using western blotting.

### Luciferase assay

As described previously [11, 59], cells were co-transfected with p21-Luc, pCMV-β-gal (Promega) and the indicated plasmids. The luciferase activity was measured two days post-transfection using a LB9507 luminometer and a luciferase assay reagent (Promega); the values were normalized to β-galactosidase activity.

### Subcellular fractionation

Cell fractionation was performed according to the manufacturer's instructions (ProteoJET Cytoplasmic and Nuclear Protein Extraction Kit, Fermentas; or NE-PER Nuclear Protein Extraction Kit, Pierce; or Cytoplasmic and Nuclear Protein Enrichment Kit, Amresco).

## SUPPLEMENTARY FIGURES


