# Supplementary material for: UBE4B targets phosphorylated p53 at serines 15 and 392 for degradation
Source: Oncotarget. 2015 Dec 10;7(3):2823–36. doi: 10.18632/oncotarget.6555 (PMC4823074; doi:10.18632/oncotarget.6555)
Supplement: Supplementary file 1 [file oncotarget-07-2823-s001.pdf]

SUPPLEMENTARY FIGURES

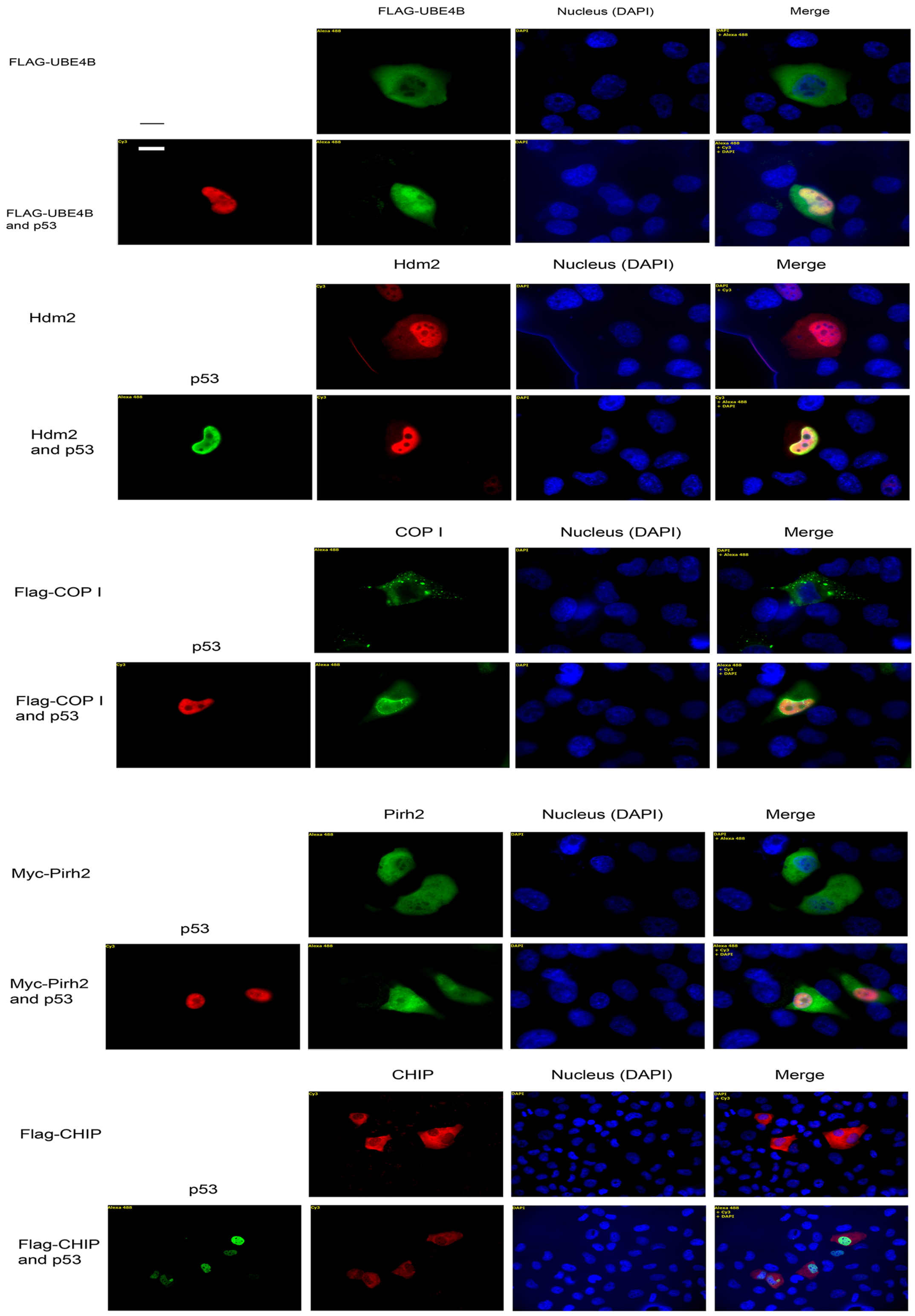

Supplementary Figure S1: Immunofluorescence analysis of the subcellular localization of p53 and various E3 ligase proteins. H1299 cells were transfected with plasmids expressing UBE4B, Hdm2, Cop1, Pirh2 and CHIP, or in combination with the p53 expression plasmid as indicated. After 40 hours, the cells were fixed and stained with the indicated antibodies. DAPI was used for DNA staining (blue). The same magnification was used for all the images and the scale bar for 10 micrometer was indicated in the second top left image.

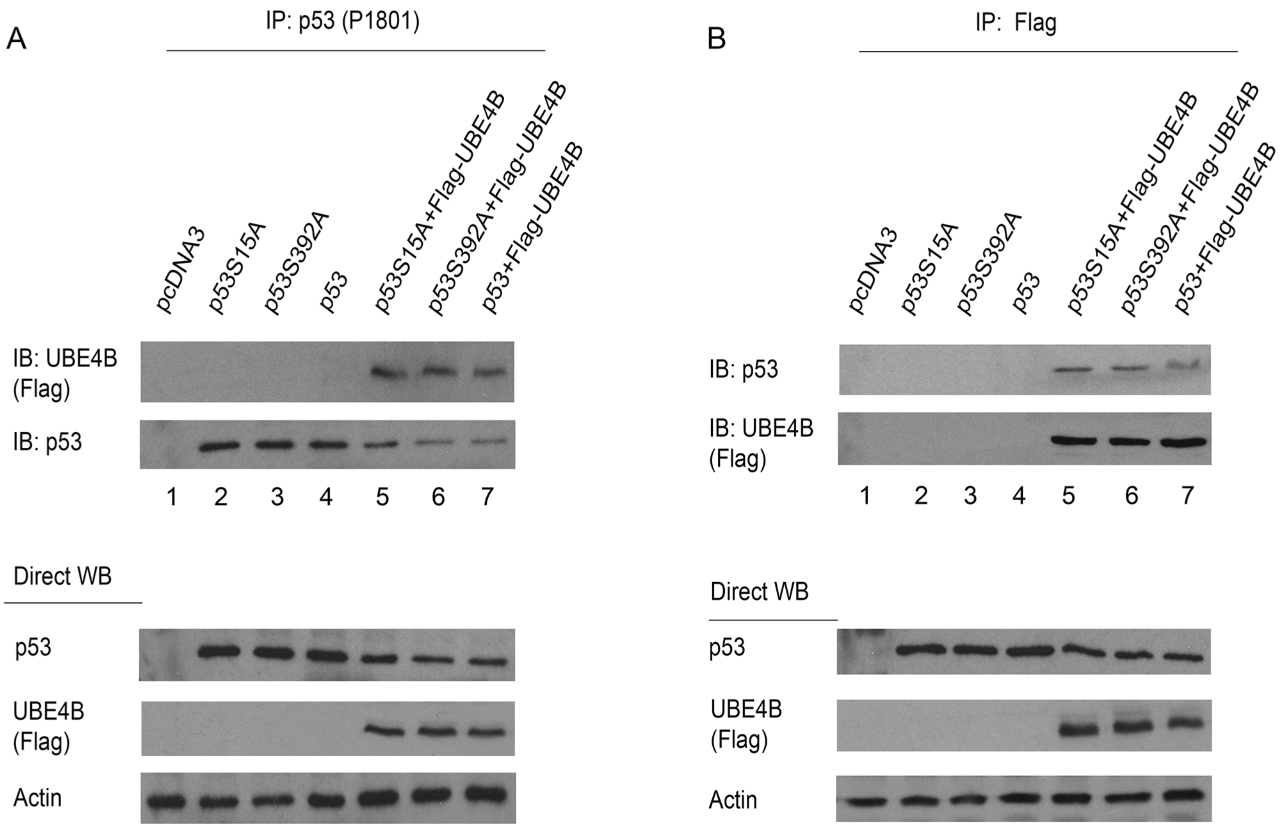

Supplementary Figure S2: UBE4B interacts with p53 and p53 variants. (A). H1299 cells were transfected with plasmids expressing p53, p53S15A, p53S392A, or in combination with Flag-UBE4B, immunoprecipitated with an anti-p53 specific antibody (Pab1801), and analyzed by western blots with anti-Flag (M2) for UBE4B and FL-393 (Santa Cruz) for p53 as indicated. Direct western blots for p53, p53 variants and UBE4B are shown in the lower panels. **(B)**. Similar to **(A)** except that cell extracts were immunoprecipitated with anti-Flag, and immunoblotted with an anti-p53 specific antibody (Pab1801) and anti-Flag as indicated. Direct western blots for p53, p53 variants and UBE4B are shown in the lower panels.
